# Detection of *Varicella-Zoster* Virus DNA in the Iris of a Zoster Sine Herpete Patient

**Published:** 2006-09

**Authors:** Mei-Ju Chen, Ko-Hua Chen, Yu-Mei Chung, An-Fei Li, Ching-Kuang Chou, Wen-Ming Hsu

**Affiliations:** 1 *Department of Ophthalmology, Taipei Veterans General Hospital, Taiwan (ROC);*; 2 *National Yang-Ming University, 201, Section II, Shih-Pai Road, Taipei, Taiwan (ROC);*; 3 *Division of Medical Engineering, National Health Research Institutes, Taipei, Taiwan (ROC)*

**Keywords:** Zoster Sine Herpete, polymerase chain reaction, iris, iridocyclitis

## Abstract

A 55-year-old man presented with unilateral iridocyclitis and elevated intraocular pressure (IOP) in his right eye. *Varicella-zoster* virus (VZV) DNA was detected by polymerase chain reaction (PCR) in the iris of a patient of Zoster Sine Herpete. No symptoms or signs of herpes zoster like neuralgia or cutaneous eruptions on forehead were noted. His iridocyclitis was treated and responded well with systemic and topical acyclovior as well as topical steroid. However, the marked elevated IOP could not be controlled by maximal dosage of anti-glaucomatous medicine. The patient underwent trabeculectomy to control his IOP. Samples of aqueous humor and iris tissue were obtained and VZV was checked by PCR. VZV virus DNA was detected from samples of the aqueous humor and iris tissue patient of Zoster Sine Herpete by PCR analysis.

## INTRODUCTION

Zoster Sine Herpete is a rare ocular disease characterized by herpes zoster ophthalmicus without cutaneous eruptions (1, 2). The diagnosis is mainly based on the clinical characteristics and serological evidence (1). Identification of *Varicella-zoster* virus (VZV) DNA in the aqueous humor samples by polymerase chain reaction (PCR) has been shown useful for rapid diagnosis of Zoster Sine Herpete, but the ocular tissues have never been tested in such patients (2, 3). Here we report the detection of VZV DNA using PCR in the iris tissue of a Zoster Sine Herpete patient.

## CASE REPORT

**Figure 1:**
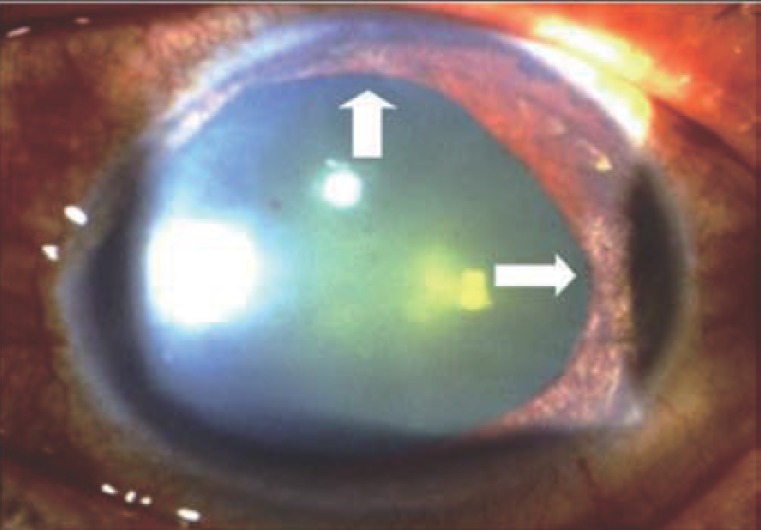
The right eye of a 55-year-old male patient under Slit lamp microscopy examination showed a fixed and dilated pupil with sectoral iris atrophy on 6 and 9 o’clock (arrows).

An otherwise healthy 55-year-old man with a painful right eye and elevated intraocular pressure (IOP) was referred to our department. He had been suffered from painful right eye off and on for 6 years. No symptoms or signs of herpes zoster like neuralgia or cutaneous eruptions on forehead were noted in the past. On his first visit, his visual acuity was 20/80 in the right eye and 20/20 in the left eye. IOPs were 45 mmHg in the right eye and 15 mmHg in the left eye. Slit-lamp microscope examination showed marked ciliary congestion of his right conjunctiva, 2+ cells and 3+ flares in the anterior chamber and a few white mutton-fat keratitic precipitates in the right eye. The pupil was fixed and dilated with sectoral iris atrophy on 6 and 9 o’clock (Figure 1). There was moderate cataractous change in the right lens. Posterior segments were unremarkable in both eyes. Chest x-ray and blood count were normal. The serologic tests showed negative IgM titers but IgG titers to herpes simplex virus (1:16) and VZV (1:32) were positive.

On suspicion of Zoster Sine Herpete, his iridocyclitis was treated with oral acyclovior 200 mg four times per day, topical acyclovior ointment five times daily and 1% prednisolone acetate eye drops four times daily. The iridocyclitis subsided within 3 weeks. However, a persisted elevated IOP of more than 35 mmHg was noted despite maximal tolerated dosage including oral acetazolamide 500 mg four times daily, 0.5% timolol gel once daily and 0.2% brimonidine three times daily. The patients underwent trabeculectomy and mitomycin-C soaking (0.02%, 3 minutes) 6 weeks later to control his IOP. The aqueous (0.1 ml) and iris sample were obtained from the right eye during the surgery and stored at -80°C immediately. After the informed consent was obtained, these samples were sent fro PCR test for VZV.

The PCR method was the same with the previous report (2). In brief, DNA was extracted from ocular samples with an Easy-DNATM kit (Invitrogen Corp, Carlsbad, CA, USA) according to the manufacturer’s recommendations. Amplification of the human β-actin gene served as an internal positive extraction control. The primer sequences for VZV were B1-5’-TTCAGCCAACGCCAATAAA-3’ and B2-5’-GACGCGCTTAACGGAAGTAAC-3’. These primers were used to amplify a 642-base-pair target sequence that was incorporated in the EcoR1-D fragment of the VZV genome.

Trabeculectomy reduced his IOP to approximately 12 mmHg successfully. Topical steroid was tapered gradually and discontinued after 2 months. The IOP remained in the normal range throughout the follow-up for 6 months.

## RESULTS

**Figure 2:**
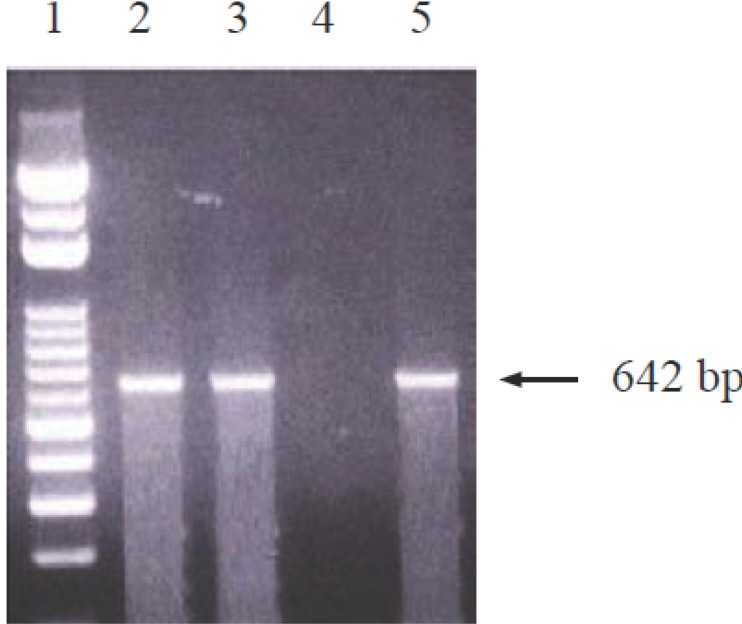
Gel electrophoresis profiles of polymerase chain reaction (PCR) products. Lane 1 is a 100-base pair (bp) ladder molecular weight standard (Protech Technology, Taipei, Taiwan). Lane 2 is aqueous humor sample from our patient showing specific 642-bp amplification of the *Varicella-zoster* virus band. Lane 3 is iris sample from our patient demonstrating the same positive band. Lane 4 is a negative control (no template added to the PCR), and Lane 5 is a positive control (sample from a patient with typical herpes zoster ophthalmicus).

Figure 2 showed a positive amplified band of VZV DNA in samples of aqueous humor as well as iris tissue.

## DISCUSSIONS

VZV is a well-recognized etiologic agent for iridocyclitis (4). To diagnose a zoster iridocyclitis without the cutaneous eruptions, zoster sine herpete, is usually difficult, if without serological evidences. It has been reported that VZV could be detected using PCR in aqueous humor (3), vitreous and lens nucleus (2) in cases of zoster sine herpete. So we tried to demonstrate VZV in the iris of a zoster sine herpete patient.

VZV antigen in the iris of an iridocyclitis patient has been demonstrated in immunohistological studies (5). It was shown that VZV antigen-positive cells were found in the vascular endothelium of the iris stroma. However, this method usually takes longer time to perform than PCR does. Thus it is less valuable then PCR in clinical diagnosis and treatment. To prevent the possible contamination of VZV virus from the aqueous humor, we washed the iris specimen with balanced salt solution (BSS) for three times. The BSS samples were sent for the same PCR test for VZV DNA and none of them was shown positive result.

The present case showed typical figures of iridocyclitis of herpes zoster ophthalmicus, including flare and cells in anterior chamber, mutton fat corneal precipitates, sectoral iris atrophy and marked increased IOP. These clinical features as well as the PCR data made the diagnosis of zoster sine herpete in this case. To our knowledge, there has been no previous report of PCR detection of VZV from the iris of cases of Zoster Sine Herpete and this is the first reported case.
